# Homogeneous Adaboost Ensemble Machine Learning Algorithms with Reduced Entropy on Balanced Data

**DOI:** 10.3390/e25020245

**Published:** 2023-01-29

**Authors:** Mahesh Thyluru Ramakrishna, Vinoth Kumar Venkatesan, Ivan Izonin, Myroslav Havryliuk, Chandrasekhar Rohith Bhat

**Affiliations:** 1 Department of Computer Science and Engineering, Faculty of Engineering and Technology, JAIN (Deemed-to-be University), Bangalore 562112, India; 2 School of Information Technology and Engineering, Vellore Institute of Technology, Vellore 632014, India; 3 Department of Artificial Intelligence, Lviv Polytechnic National University, 79013 Lviv, Ukraine; 4 Institute of Computer Science and Engineering, Saveetha School of Engineering (SIMATS), Chennai 602105, India

**Keywords:** machine learning, entropy, breast cancer, ensemble methods, precision

## Abstract

Today’s world faces a serious public health problem with cancer. One type of cancer that begins in the breast and spreads to other body areas is breast cancer (BC). Breast cancer is one of the most prevalent cancers that claim the lives of women. It is also becoming clearer that most cases of breast cancer are already advanced when they are brought to the doctor’s attention by the patient. The patient may have the evident lesion removed, but the seeds have reached an advanced stage of development or the body’s ability to resist them has weakened considerably, rendering them ineffective. Although it is still much more common in more developed nations, it is also quickly spreading to less developed countries. The motivation behind this study is to use an ensemble method for the prediction of BC, as an ensemble model aims to automatically manage the strengths and weaknesses of each of its separate models, resulting in the best decision being made overall. The main objective of this paper is to predict and classify breast cancer using Adaboost ensemble techniques. The weighted entropy is computed for the target column. Taking each attribute’s weights results in the weighted entropy. Each class’s likelihood is represented by the weights. The amount of information gained increases with a decrease in entropy. Both individual and homogeneous ensemble classifiers, created by mixing Adaboost with different single classifiers, have been used in this work. In order to deal with the class imbalance issue as well as noise, the synthetic minority over-sampling technique (SMOTE) was used as part of the data mining pre-processing. The suggested approach uses a decision tree (DT) and naive Bayes (NB), with Adaboost ensemble techniques. The experimental findings shown 97.95% accuracy for prediction using the Adaboost-random forest classifier.

## 1. Introduction

The number of records and database sizes holding medical data are both expanding. Various databases are constantly maintained with medical data because of measurements, tests, prescriptions, and other procedures. Traditional methodologies cannot analyze and search for intriguing patterns and information within this enormous amount of data [1]. In order to find useful information in massive data repositories, there is a growing need for cutting-edge methods and technologies. Cancer has emerged as one of the deadliest illnesses in recent years. Women all across the world are particularly susceptible to breast cancer. In the field of bioinformatics or medical research, the accurate diagnosis of certain vital information is a significant problem [2]. Countless websites, hospitals, research facilities, and diagnostic organizations all have access to enormous amounts of medical diagnostic data. To automate and expedite disease diagnosis, categorization is merely a supplementary step. Breast tumors, which can be benign or malignant, are most frequently caused by an overgrowth of the cells lining the breast ducts. A benign tumor forms when the cells grow incorrectly. The most prevalent type of benign breast tumor is a fibro adenoma [3]. This could need to be surgically removed to establish the diagnosis, and further therapy might not be required. Malignant tumors’ cancerous cells have the capacity to spread outside of the breast if untreated. If caught early enough, breast cancer is usually curable.

A lump or clump, changes in breast or nipple size or shape, changes in breast or nipple color, the possibility of nipple discharge, breast enlargement or thickness, or even persistent discomfort are some of the signs of breast cancer [4,5]. During a screening exam, it can be caught early using mammography or a portable cancer detection tool. According to the cancer stage, the tissues of breast cancer evolve as the condition worsens. The breast cancer stage (I–IV) indicates how far cancer has extended in that patient [6].

Stage I: The tumors grow slowly and are unlikely to spread. This stage can often require surgery to cure.

Stage II: The tumors grow and spread very little, but this stage may come back after the treatment.

Stage III: The tumors rapidly divide the cells’ growth, but no dead cells are found. This stage grows quickly.

Stage IV: The tumors are actively dividing, and they have both growth and dead tissues. In this stage, tumors can grow and spread quickly.

Breast cancer can take many different forms. There are many different ways to categorize and forecast this condition [7].

It is well known that ensemble classifiers outperformed single classifiers in most studies conducted over the past ten years. The motivation behind this study is to use a homogeneous classifier, as it combines one base method with at least two alternative configurations or variants, or an ensemble that combines one base learning model with one meta ensemble, such as boosting.

The main contributions of this study are the following:

(1) We improved the quality of data in pre-processing using the SMOTE technique.

(2) We used the average splitting technique to randomly divide the dataset into smaller subsets and then individually model each division using the different classifiers.

(3) Utilizing the accuracy ranking technique to keep the best base models and discard the worst ones will help the suggested homogeneous ensemble technique perform better.

The article is organized as follows. The following section provides a review of the literature and related work carried out in breast cancer detection. The third section shows the proposed model, and the next section provides the performance analysis of different ML algorithms and ensemble methods against various performance factors, namely precision, recall, and f1 measures.

## 2. Related Works

Some women may have an increased chance of developing breast illness due to genetics, lifestyle, weight, radiation, and regeneration factors. Due to the condition, when identified quickly, the patient can be rescued due to the advancements in the treatment of malignant developments [8]. Fit imaging and ongoing organizing have been demonstrated to advance image objectives and painful depictions.

Breast electrography, in addition to traditional ultrasonography (US) as well as mammography, provides information on breast abnormalities. It provides a non-invasive assessment of a lesion’s stiffness [9]. The major findings suggest that it can improve on USG’s specificity and predictive value when describing breast masses. Any injury is visible in mammography or USG for the reason that the sore’s general thickness and echogenicity are different from the surrounding breast tissue. Breast illness prediction using a constructed hereditary calculation-based technique was offered as a framework, and it was noted that the prediction of breast cancer growth is still an unexplored area. In this article [10], various AI calculations are used to forecast the location of the breast illness. Decision trees, irregular woods, SVMs, brain organizations, direct models, Adaboost, and pure Bayesian methods are used for forecasting. An engineering technique is used to improve the accuracy of predicting breast disease. The GA-based weighted normal total methodology for direct information collecting is a recently developed method that circumvents the limitations of the traditional weighted normal technique [11]. A few models are predicted using the weighted normal technique with consideration for hereditary calculations.

SVM, MLP, and NN are just a few of the ML algorithms that are used to evaluate the Wisconsin Diagnostic Dataset [12]. The aforementioned dataset includes highlights found from FNA test sweeps on a bosom growth. The informative index is divided into two parts for ML calculations: 70% for preparation and 30% for testing [13]. Their findings revealed that all of the ML computations introduced a better performance on the twofold order of carcinomas, such as determining whether growth is safe or harmful. In this approach [14], the factual solutions to the arranging problem are also agreeable. It is advised to use a CV process, such as K-fold cross-validation, to validate this review’s outcomes [15]. This incrementing will help identify the key ideal hyperparameters for ML calculations and a more precise percentage of model expectation execution. In this paper [16], ML tactics that improve indicative precision are examined. CART, RF, and K-nearest neighbors were considered as techniques. The UCI (University of California Irvine) machine learning repository currently maintains 488 datasets of various characteristics and was used to obtain the dataset.

It is frequently noticed that the KNN computation performs significantly better than other correlation-related methods [17]. The K-nearest neighbor model is the most trustworthy. Models for grouping, such as random forest and boost tree, demonstrate a comparative precision [18]. Therefore, the most reliable classifier may be used to identify growths so that a fix can be discovered at an early stage. Breast illness can be diagnosed using several AI methods and blood test data to rule out cancer early. Four different AI computations are used in this article to identify cancer early on [19].

Malignant tumors are cancerous, while benign tumors are not, and are typically less dangerous. However, they do not invade other tissues or organs [20]. They can develop to great proportions, which may result in pressure on the region around the tumor, which may result in discomfort and other complications. If medically removed, they are likely to remain in the same location, developing slowly and preserving the defined borders around the perimeter of the tumor [7]. Malignant tumors are malignant and can metastasize or spread to new organs and tissues to form new tumors. Primary malignant tumors disseminate to auxiliary locations during metastasis [21]. The liver, lungs, brain, and bones are frequently the sites of metastases that are established when cancer cells separate from the tumor and travel through the blood or lymphatic system [22]. Malignant tumors must, therefore, be treated right away to prevent metastasizing. Depending on how far along the cancer is, the typical course of treatment includes surgical removal, radiotherapy, chemotherapy, or a combination of these.

We have developed the AdaBoost homogeneous ensemble strategy in this study and used this novel method to develop an accurate, automatic prediction model that can distinguish benign breast cancers from malignant ones. These forecasts aim to categorize patients into benign and malignant groups, helping individuals with benign breast tumors to avoid or reduce the amount of invasive treatments they may need to perform. The suggested strategy enables us to utilize a number of ensemble approaches simultaneously to enhance the performance of the prediction system.

## 3. Proposed Methodology

**Figure 1:**
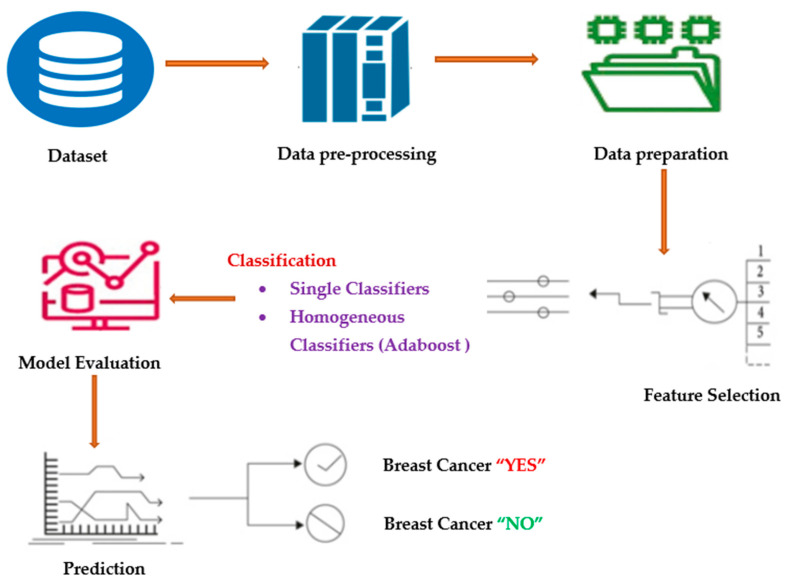
Process diagram for the proposed method.

The Breast Cancer Wisconsin (diagnostic) dataset was obtained via Kaggle. Ten independent variables—”Sample code number,” “Clump Thickness,” “Uniformity of Cell Size,” “Uniformity of Cell Shape,” “Marginal Adhesion,” “Single Epithelial Cell Size,” “Bare Nuclei,” “Bland Chromatin,” “Normal Nucleoli,” and “Mitoses”—are considered to be “X,” and one dependent variable—”Y”—consists of class labels [23]. The dataset has roughly 137 samples. The data is separated into two groups: X contains all of the input variables, while Y contains the class label, which serves as the output variable [24]. The proposed methodology is depicted in Figure 1.

### 3.1. Data-Preprocessing

A class imbalance occurs when there is an unbalanced distribution of classes in a dataset, meaning that there are many more data points in the negative class (majority class) than in the positive class (minority class). The classifier model’s performance will suffer if the skewed data are not corrected beforehand. The majority of the predictions will match the majority class, while the minority class features will be treated as data noise and ignored. The model will have a significant bias as a result. As mentioned, to achieve more accurate classification model results, the data are separated into training and test portions, with the training data comprising 70% of the total dataset, and the test portions comprising 30%.

Many classification algorithms strive to collect only pure examples for learning and make the boundary between each class as clear as possible to improve the prediction. Most classifiers find it significantly more difficult to learn how to classify synthetic cases close to the boundary than those far from it. Based on these results, the authors present an enhanced pre-processing method (A-SMOTE) for imbalanced training sets [25]. SMOTE uses a k-nearest neighbor method to generate synthetic data. SMOTE begins by randomly selecting data from the minority class, after which the data’s k-nearest neighbors are determined [26]. The k-nearest neighbor selected at random and the random data would then be combined to create synthetic data. The SMOTE method is explained in the section below.

Step A: Using Equation (1), a synthetic instance is created:

(1) N=2∗(r−z)+z,

where r is the majority class samples, *z* is the minority class samples and N is the newly created synthetic instance.

Step B: The below mentioned steps are carried out to remove the outlier; that is, noise.

If, $\hat{S}$ = {${\hat{S}}_{1}$, ${\hat{S}}_{2}$, ${\hat{S}}_{3}$, …. ${\hat{S}}_{n}$} is a new instance received by Step A, then we will calculate the distance among ${\hat{S}}_{i}$ with the original minority $S_{m},Min_{Rap}({\hat{S}}_{i}$, ${\hat{S}}_{m})$ defined using Equation (2).

(2) $$Min_{Rap}({\hat{S}}_{i},\;{\hat{S}}_{m})=\sum_{k=1}^{z}\sum_{j=1}^{M}\sqrt{{\left({\hat{S}}_{i}{}^{\left(j\right)}-S_{mk}{}^{\left(j\right)}\right)}^{2}},$$

where:

$Min_{Rap}({\hat{S}}_{i}$, ${\hat{S}}_{m})$ are samples of rapprochement and, as per Equation (2), *L* is calculated using Equation (3).

(3) $$L=\sum_{i=1}^{n}(Min_{Rap}\;\left({\hat{S}}_{i},\;S_{m}\right)),$$

Step C: Calculate the distance between ${\hat{S}}_{i}$ and every original majority $Sa,Maj_{Rap}({\hat{S}}_{i}$,$S_{a}$), described using Equation (4).

(4) $$Maj_{Rap}({\hat{S}}_{i},S_{a})=\sum_{i=1}^{r}\sum_{j=1}^{M}\sqrt{{\left({\hat{S}}_{i}{}^{\left(j\right)}-S_{al}{}^{\left(j\right)}\right)}^{2}},$$

$Maj_{Rap}({\hat{S}}_{i}$,$S_{a}$) are samples of rapprochement and, as per Equation (4), *H* is computed using Equation (5).

(5) $$H=\sum_{i=1}^{n}(Maj_{Rap}\;\left({\hat{S}}_{i},\;S_{a}\right))$$

Entropy is nothing but the measure of disorder. Claude E. Shannon used the following Equations (6) and (7) to put this link between probability and heterogeneity or impurity into mathematical form:

(6) $$H\left(X\right)=-\sum \left(p_{i}*log_{2}p_{i}\right)$$

(7) $$\mathrm{Entropy}\;(p)=-\sum_{i=1}^{N}p_{i}*log_{2}p_{i}$$

**Figure 2:**
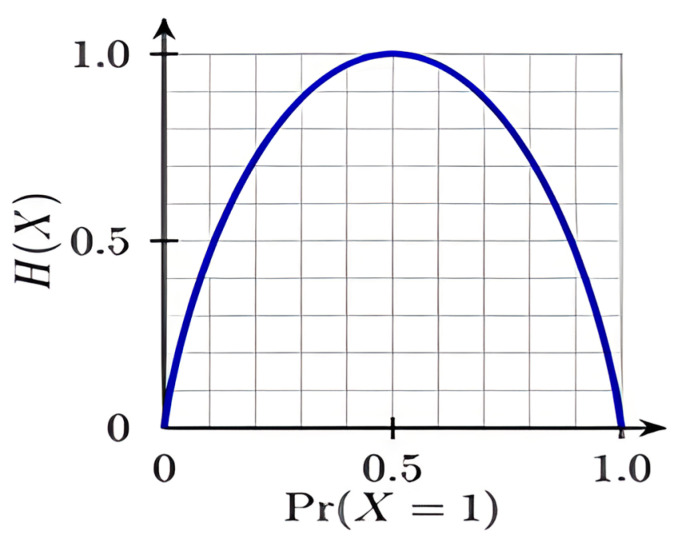
For a Bernoulli trial (X = {0,1}), the graph of entropy vs. Pr(X = 1). The highest H(X) = 1 = log(2).

The likelihood of a category’s uncertainty or impurity is given as the log to base 2 × (pi). The number of potential categories is indicated by the index *i*, and since our issue is a binary categorization, *i* = 2 in this instance. The graph below (2) shows how a symmetric curve represents his equation. The probability of the occurrence is plotted on axis X, and the heterogeneity or impurity H is shown on axis Y. The sample graph of entropy is shown in Figure 2.

### 3.2. Adaboost Classifier

Adaboost enables the fusion of many “weak classifiers” into a single, so-called “strong classifier”. These trees are often referred to as “Decision Stumps.” By giving each data point the same weight, this method builds a model. The improperly categorized points are subsequently given more weight. All points with higher weights are given more significance in the following model. The models will keep being trained until a reduced error is obtained. The pseudo code of the Adaboost classifier is depicted in Algorithm 1. Adaboost and LogitBoost, as depicted in Algorithms 1 and 2, are the two boosting algorithms that are utilized to prompt the ADTree variations discussed in the following section.
**Algorithm 1:** Adaboost classifier—pseudo codeInput: Let D be the dataset that includes {(a_1_,b_1_), (a_2_,b_2_), ….. (a_m_, b_m_)}; Let λ be the learning (base) algorithm     Let T be the total No. of learning rounds.Process:  D_1_(i) = 1/m   for time = 1, …, *T*;h_t_ = λ (D, D_t_); weak learner is trained with Distribution D_t_∈_t_ = $PrPr_{i\sim D_{t}}$[ $h_{t}$(a_i_≠b_i_)]; Error measure (entropy)$\propto_{t}$ = $\frac{1}{2}$ ln ($\frac{1-\in t}{\in t});\%\;determine\;the\;weigth\;of$ h_t_D_t+1_(i) = $\frac{D_{t}\left(i\right)}{Z_{t}}$ ∗$\{\begin{array}{c} \exp\left(-\propto_{t}\right)\;if\;h_{t}\left(a_{i}\right)=b_{i}\\ \exp\left(\propto_{t}\right)\;if\;h_{t}\left(a_{i}\right)\neq b_{i} \end{array}$
              = $\frac{D_{t}\left(i\right)\exp(-\propto_{t}y_{t}h_{t}\left(a_{i}\right)}{Z_{t}}$
Outcome: H(a) = sign ($\sum_{t=1}^{T}\propto_{t}h_{t}\left(b\right)$


Like the Adaboost, the LogitBoost also starts off with the uniform weight distribution. Additionally, it records the regression value G($x^{\left(i\right)}$) and the probability prediction of the positive class p($x^{\left(i\right)}$) at each boosting iteration.
**Algorithm 2**: LogitBoostInput: Dataset (Training)  1. Initialize: $b^{\left(i\right)*}$ = (*b*^(*i*)^ + 1)/2, $w_{i}=\frac{1}{n},$ G($a^{\left(i\right)}$) = 0, and p($a^{\left(i\right)}$) = 0.5  2. For time = 1…..,T     2.1a Calculate the working response as well as weights $z_{i}$ = $\frac{b^{\left(i\right)*}-p\left(a^{\left(i\right)}\right)}{p\left(a^{\left(i\right)}\right)\left(1-p\left(a^{\left(i\right)}\right)\right)}$,          
$w_{i}=p\left(a^{\left(i\right)}\right)*\left(1-p\left(a^{\left(i\right)}\right)\right)$
     2.1b Fit $g_{1}\left(a\right)$ using weights $w_{i}$
     2.1c Update G($a^{\left(i\right)}$) ← G($a^{\left(i\right)}$) + $\frac{1}{2}g_{t}\left(a^{\left(i\right)}\right)$ and $p\left(a^{\left(i\right)}\right)=\frac{\exp\left(G\left(a^{\left(i\right)}\right)\right)}{\exp\left(G\left(a^{\left(i\right)}\right)\right)+\;\exp\left(-G\left(a^{\left(i\right)}\right)\right)}$
Output: G(a) = $\sum_{t=1}^{T}g_{1}\left(a\right)$


At the beginning of each boosting procedure, the working response as well as weight distribution are changed. The weighted least squares regression issue is fitted with a regression function.

### 3.3. Alternating Decision-Tree (ADTree)

A ML approach for classification is ADTree. It generalizes decision trees (DTs) and is linked to boosting. A set of DT nodes representing a predicate condition and prediction nodes that will store a single integer make up an ADTree [27]. The Real Adaboost is implemented in Algorithm 3 to learn an ADTree model. The precondition P and base conditions C remain constant during the induction as two significant variables. The division of the input space greatly affects any decision tree’s quality.

In step 1.1 of Algorithm 3, all the training samples are given an equal weight by setting $W_{i,t}$ = 0 = 1/n. The training sample as well as the boosting step are represented by the symbols I and t, respectively. Additionally, the precondition true, which is going to define the complete input space, is initialized as part of the precondition set P. With the root decision rule, a prior classifier is created by deriving a second prediction value, $\alpha_{0}$ from the ratio of positive to negative training data. $W_{+}$(c) stands for the total weight of the positive samples that meet condition c, and $W_{-}$ (c) for the negative samples. Before starting a fresh boosting procedure, the training data set is reweighted in accordance with this root decision rule (step 1.3).
**Algorithm 3:** ADTree with AdaboostInput: Training Dataset -D  1. Process of Initialization       1.a Set $w_{i,t}$ = 0 = 1/n $\forall i\;and\;P_{t=1}$ = {true}      1.b First DT rule $r_{0}$(x): { if (true) then $[if\;\left(true\right)\alpha_{0}$ = $\frac{1}{2}$ ln ($\frac{W+\left(true\right)}{W-\left(true\right)})\;else\;0]$ else 0}1.c Update $w_{i,t}$ = 1 = $w_{i,t}$ = 0 exp$(-r_{0}$
$\left(x^{\left(i\right)})\;y^{\left(i\right)}\right)$
2. Do it again for boosting cycle t = 1:T2.1 For every pre-condition $C_{1}\in \;P_{t}$ and each condition $C_{2}\in C,\;evaluate$
Z$(c_{1},\;c_{2})=2(\sqrt{W+\left(c_{1}\cap c_{2}\right)W-\left(c_{1}\cap c_{2}\right)}$ + $\sqrt{W+\left(c_{1}\cap \neg c_{2}\right)W-\left(c_{1}\cap \neg c_{2}\right)}$+ W($\neg c_{2})$
    2.1 Compute $\alpha_{t}^{+}$ and $\alpha_{t}^{-}$ for the selected $c_{1}^{*}\;and\;\alpha_{1}^{*}$ that minimizes Z with δ = 1 $\alpha_{t}^{+}$ = $\frac{1}{2}$ ln ($\frac{W+\left(c_{1}^{*}\;\cap \;c_{2}^{*}\;\right)+\delta \;\;}{W-\left(c_{1}^{*}\;\cap \;c_{2}^{*}\;\right)+\delta \;}$), $\alpha_{t}^{-}$ = $\frac{1}{2}$ =l n($\frac{W+(c_{1}^{*}\;\cap \neg c_{2}^{*}\;)+\delta \;\;}{W-(c_{1}^{*}\;\cap \neg c_{2}^{*}\;)+\delta \;}$)     2.2 Update $P_{t+1}$: $P_{t}\;\cup$ { $c_{1}^{*}\;\cap \;c_{2}^{*}$, $c_{1}^{*}\;\cap \neg c_{2}^{*}\}$
    2.3 Update $W_{i,t+1}$ = $W_{i,t}$ exp(- $r_{t}$ ($x^{\left(i\right)})\;y^{\left(i\right)})$
Output: F(x) = $\sum_{t=1}^{T}r_{1}\left(x\right)$


### 3.4. Reduced Error Pruning Tree (REPTree)

Poor pruning phase performance has been seen to hinder the top-down induction of DTs. For instance, despite the truth that the accuracy of the generated tree does not increase, it is known that its size increases linearly with the sample amount. The errors are minimized. The REPTree method is based on the idea that a variance-induced error can be reduced by computing the information gain using entropy and backfitting.

### 3.5. Naïve Bayes (NB) Classifier

In the naive Bayesian technique, the categorized data are presented in two steps. The initial step of utilizing the training input data entails assessing the probability distribution’s parameters. The test dataset is categorized in the second stage according to the highest posterior probability. The pseudo code for the NB classifier is displayed here.

### 3.6. Random Forest (RF) Classifier

The training data are used to build many decision trees, which is how RF functions. Every tree in a classification situation suggests an output as a class, and the class with the greatest number of outputs is ultimately chosen. The amount of trees needed for construction must be given. Such a method for aggregating data is called random forest (RF). This technique is used to lower the variation in the results, a crucial metric.

### 3.7. CART

Breiman et al. were the first to present the CART algorithm. CART is produced with the use of Hunt’s algorithm. It can process categorical and continuous attributes to create a DT. Additionally, it takes into account missing data and builds the DT using the Gini Index as an attribute selection criterion [28]. CART creates binary trees by splitting the given datasets (training set) into binary segments. The probabilistic assumptions for ID3 and C4.5 do not use the Gini Index. The CART method uses cost-complexity pruning to eliminate erratic branches from the DT to improve classification accuracy.

### 3.8. Homogeneous Adaboost Technique

The mentioned algorithms, namely ADTree, NB classifier, RF classifier, REPTree and CART classifiers are executed as individual classifiers on the given dataset, and their performance against precision, recall, f1 measure and accuracy are compared. In the next stage, the Adaboost classifier is combined with each of these individual classifiers, the same is referred to as a homogeneous Adaboost classifier and the performance is recorded against different metrics as mentioned. Then, finally, the model that provides the best performance is recommended for deployment in the early detection of breast cancer.

## 4. Results and Discussion

The various ML techniques of naive Bayes, ADTree, REPTree, CART and RF are used in the dataset as separate classifiers. In the following section, a comparison of their performances is made using different metrics [29]. The f1 measure, recall, precision, and accuracy are computed using Equations (8)–(11).

(8) $$Precision=\frac{TPs}{TPs+FPs}$$

(9) $$recall=\frac{TPs}{TPs+FNs}$$

(10) $$F1\;Score=2*\frac{precision*recall}{precision+recall}$$

(11) $$Accuracy=\frac{TNs+TPs}{TNs+TPs+FPs+FNs}$$

The mean absolute error (MAE), root mean squared error (RMSE), relative absolute error (RAE) and relative squared error (RRSE) are computed using Equations (12)–(15).

(12) $$MAE=\sum_{i=1}^{p}|\;r_{i}-r_{i}{}^{T}|$$

(13) $$RMSE=\sqrt{\frac{\sum_{r=1}^{p}{\left(r_{i}-r^{T}{}_{i}\right)}^{2}}{p}}$$

(14) $$RAE=\frac{\sum_{r=1}^{p}{\left(r_{i}-r^{T}{}_{i}\right)}^{2}}{\sum_{r=1}^{p}{\left(r_{i}-{\bar{r}}_{i}\right)}^{2}}$$

(15) $$RRSE=\sqrt{\frac{\sum_{r=1}^{p}{\left(r_{i}-r^{T}{}_{i}\right)}^{2}}{\sum_{r=1}^{p}{\left(r_{i}-{\bar{r}}_{i}\right)}^{2}}}$$

According to Table 1, random forest is the best model because it can be built in 2.11 s, as opposed to 58.28 s for ADTree (TTBM—time to build model).

**Figure 3:**
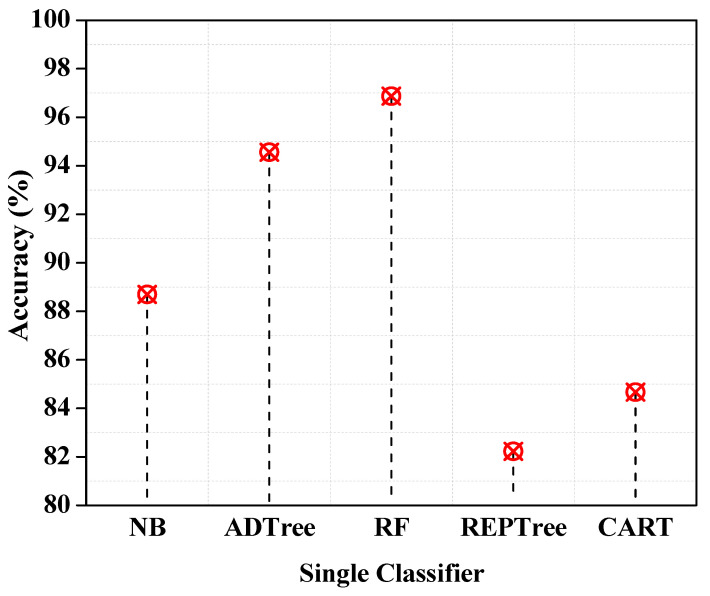
Accuracy of single classifiers.

For each classifier, the accuracy forecast is shown in Figure 3. RF has the best accuracy of 96.87% among all the classifiers described above that are currently being employed in the research. The accuracy provided by random forest is the best, and the lowest is provided by CART classifier prediction, with an accuracy of 84.67%.

**Figure 4:**
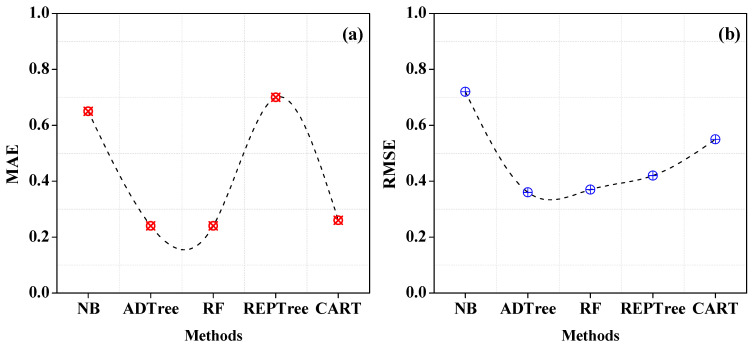
Individual classifier—error rates. (**a**) MAE of Individual Classifiers (**b**) RMSE of Individual Classifiers.

The rates of mistakes derived from each classifier are shown in Figure 4a,b. The ADTree RMSE rate value is 0.36 and the MAE rate is 0.24, respectively. This demonstrates that the prediction processes recorded few errors. However, the error rate for NB is higher, having 0.72 and 0.65 for RMSE and MAE, respectively.

The Adaboost is now an ensemble with the individual classifiers described as homogeneous classifiers, as mentioned earlier. AdaBoost-RF is the best model, as shown by Table 2, which also shows that it was built in just 8.52 s. The AdaBoost-CART, however, is the worst model because it requires 200.12 s to create. AdaBoost-NB has the lowest F1-value (0.70), whereas AdaBoost-RF has the greatest F1-value (0.98).

**Figure 5:**
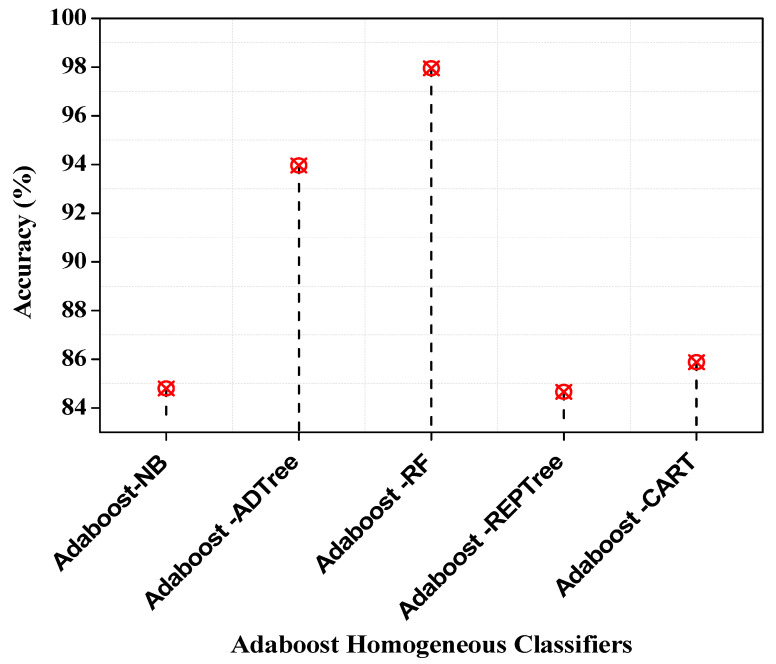
Accuracy of Adaboost homogeneous classifiers.

With an accuracy of 97.95 percent, the Adaboost-RF forecasts outperform all other classification algorithms, as shown in Figure 5. Adaboost-ADTree, on the other hand, comes in second with a prediction accuracy of 93.96%. Adaboost-REPTree offers a prediction rate of 84.66%, which is the lowest.

**Figure 6:**
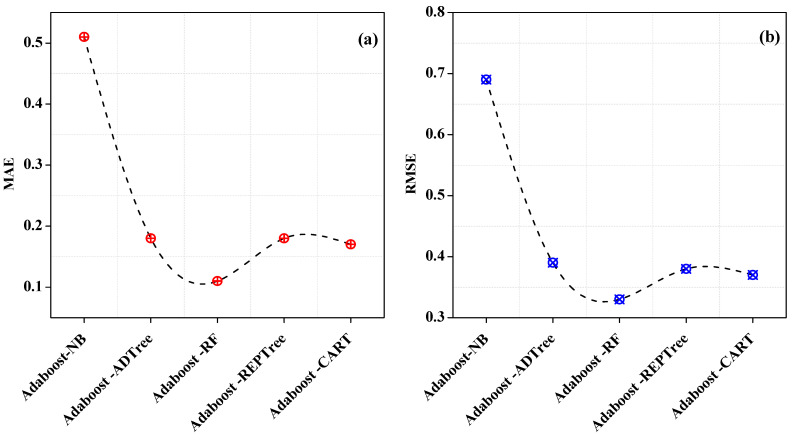
Error rates of Adaboost homogeneous classifiers. (**a**) MAE of Adaboost homogeneous classifiers (**b**) RMSE of Adaboost homogeneous classifiers.

The varying error rates that were observed are shown in Figure 6. The lowest error rates are offered by Adaboost-RF ensemble classifiers, which are 0.11 for MAE as well as 0.33 for RMSE. On the other hand, Adaboost-NB has a greater error rate, with values for MAE and RMSE of 0.51 and 0.69 that are very close to the values of the NB individual classifier.

All the performance measures reported in this study are statistically different compared to the other studies, as shown in Table 3.

In the proposed method, we estimated 95% confidence intervals (CI) based on 10 replications for different random seed numbers. Using a holdout (70–30%) validation technique, Walid Theib Mohammad et al. [33] reported a maximum classification accuracy of 97.7%, while our proposed study achieved 97.95% (95% CI: 96.5–98.6%) classification accuracy. In summary, we have achieved 0.25% and 0.59% higher accuracies compared to the second-best models by Walid Theib Mohammad et al. [33] and Chaurasia V et al. [31] (10-fold cross validation), respectively.

## 5. Conclusions

An Adaboost ensemble model has been proposed in this article for the prediction of breast cancer based on recognized feature patterns. These tools help patients and medical professionals acquire as much data as they can in the real world. The goal is to identify the most effective algorithm for properly predicting the incidence of breast cancer. This article’s main objective was to summarize all the prior and ongoing research on ML algorithms that have been used to breast cancer prediction. To deal with the class imbalance issue, the data mining pre-processing technique uses synthetic minority over-sampling technique (SMOTE). The experimental results show that 8.52 s for Adaboost-RF and 18.32 s for Adaboost-NB ensemble are the optimal times to develop the model for homogeneous ensemble classifiers. The results show that, among the homogeneous Adaboost classifiers, the AdaBoost-RF classifier has the lowest error rate of 0.13 for MAE as well as the highest error rate of 0.36 for RMSE. AdaBoost-RF outperformed all other classifiers with an accuracy rate of 97.95% in all of the overall studies in which classifier performance was tested. The dataset quality can be further improved using the recent advanced SMOTE technique and, in the future, a heterogeneous combination of the ensemble methods can be employed to achieve an improved performance.

## Figures and Tables

**Table 1:** Single classifiers—performance comparison.

Performance Metrics	NB	ADTree	RF	REPTree	CART
TTBM (s)	5.77	58.28	2.11	11.76	55.55
Accuracy (%)	88.7	94.56	96.87	82.23	84.67
F1-Score	0.3	0.85	0.84	0.83	0.81
RAE	120	56.71	76.12	76.92	65.77
MAE	0.65	0.24	0.24	0.7	0.26
RRSE	137.51	94.33	80.72	96. 79	96.44
RMSE	0.72	0.36	0.37	0.42	0.55

**Table 2:** Homogeneous (Adaboost) classifier.

Performance Metrics	Adaboost-NB	Adaboost-ADTree	Adaboost-RF	Adaboost-REPTree	Adaboost-CART
TTBM (s)	18.32	30.21	8.52	61.44	200.12
Accuracy (%)	84.8	93.96	97.95	84.66	85.88
F1-Score	0.70	0.87	0.98	0.94	0.79
MAE	0.51	0.18	0.11	0.18	0.17
RMSE	0.69	0.39	0.33	0.38	0.37
RAE	107.44	55.64	33.77	44.29	42.48
RRSE	135.62	91.12	61.33	91.33	89.66

**Table 3:** Comparison of the outcomes of the proposed study with other studies found in the literature.

Study and Year	Sampling Strategy	Accuracy
Alzubaidi A et al. 2016 [30]	70–30% training–testing	97.0%
Chaurasia V et al. 2018 [31]	10-fold cross validation	97.36%
Islam et al. 2017 [32]	10-fold cross validation	97.0%
Walid Theib Mohammad et. 2022 [33]	70–30% training–testing	97.7%
Asri et al. 2016 [34]	10-fold cross validation	97.13%
Proposed Method (Adaboost-RF)	70–30% training–testing	97.95%
	95% CI	96.5–98.6%

## Data Availability

The dataset used for the findings is included in the manuscript.
